# Efficacy of Palifermin in the Hematopoietic Stem Cell Transplant Setting

**Published:** 2013-03-01

**Authors:** Muneera Panjwani

**Affiliations:** From Yale University School of Nursing, New Haven, Connecticut

## Abstract

Palifermin is a recombinant human keratinocyte growth factor that stimulates proliferation and differentiation of epithelial cells. Palifermin’s biological activity exerts cytoprotective and healing effects that decrease cell injury caused by chemotherapy and radiation therapy. In randomized, placebo-controlled trials, palifermin significantly reduced the incidence and duration of severe oral mucositis. Based on these findings, the US Food and Drug Administration approved palifermin for patients with hematologic malignancies undergoing myeloablative therapy followed by hematopoietic stem cell transplant (HSCT). However, researchers testing the efficacy of palifermin in postapproval studies using various conditioning regimens have debated the extrapolation of palifermin dosage and dosing frequency used in the registration study as inappropriate for less mucotoxic agents. In addition, modifying the dosing intervals and frequency of palifermin has been proposed to decrease adverse events and achieve the highest clinical benefits for less mucotoxic regimens. The incidence and severity of oral mucositis vary significantly across different conditioning regimens. Hence, cost-effectiveness and the clinical benefits of palifermin among various conditioning regimens have also been debated. This article reviews the published literature on the efficacy of palifermin and makes evidence-based recommendations for the use of palifermin in the HSCT setting.

Oral mucositis (OM) is a common and debilitating side effect of myeloablative conditioning regimens administered to patients with hematologic malignancies undergoing hematopoietic stem cell transplant (HSCT). The overall incidence of mucositis in the HSCT setting is reported to be 75% to 99% (Radtke & Kolesar, 2005). Oral ulcerations cause pain when eating, drinking, swallowing, and even talking, leading to poor nutritional status and negatively impacted quality of life. Oral mucositis is also associated with an increased risk of serious infections and use of health-care resources such as the need for total parenteral nutrition (TPN), IV antibiotics, and pain medications (Schmidt et al., 2008).

For many years, the clinical care of OM has been directed toward palliation and symptom management through use of oral rinses, protective coating agents, topical anesthetics, and systemic analgesics (Blijlevens & Sonis, 2007). Unfortunately, there are many gaps in the literature, and current evidence is insufficient to support the recommendation of many of these agents for the management of OM (Stokman et al., 2006). Fortunately, better understanding of the underlying pathobiology of mucositis is shifting the focus from symptom management to prevention of OM. Palifermin (Kepivance) is a biological agent that targets the pathobiology of OM by exerting cytoprotective and regenerative effects on the oral mucosa, leading to a decreased incidence, duration, and severity of OM.

Sonis and colleagues (2007) have proposed a pathobiological model for OM consisting of five phases: initiation, upregulation, signal amplification, ulceration, and healing. Palifermin’s biological activity at each of these phases is theorized to decrease chemotherapy- and radiotherapy-induced mucosal injury. These include cytoprotective effects, modulation of the cytokine profile, and trophic or regenerative effects. The cytoprotective effects are exerted by upregulation of detoxifying enzymes, which protects against mucosal cell injury from reactive oxygen species (ROS). There is also activation of enzymes that prevents damage to DNA strands. Palifermin modulates the cytokine profile by downregulating helper T-cell type-1 proinflammatory cytokines and upregulating helper T-cell type-2 cytokines such as interleukin (IL)-4 and IL-13. Regenerative effects are induced by stimulation of migration, proliferation, and differentiation of epithelial cells; see Figure 1 (Athar & Gentile, 2009; Blijlevens & Sonis, 2007).

**Figure 1:**
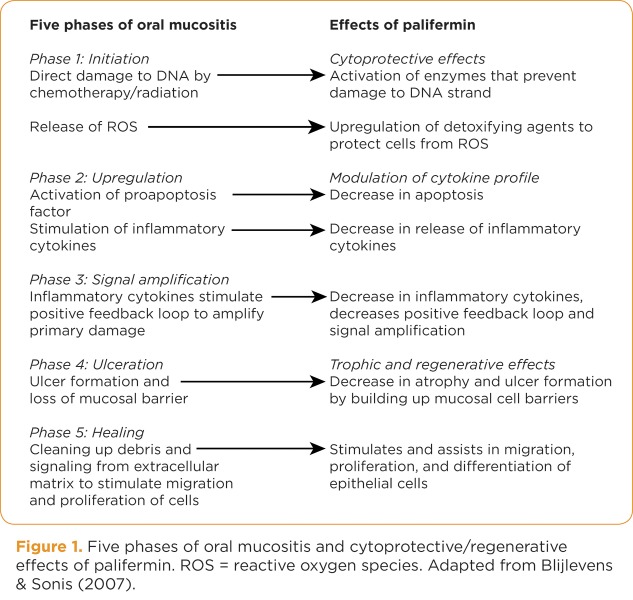
Figure 1. Five phases of oral mucositis and cytoprotective/regenerative effects of palifermin. ROS = reactive oxygen species. Adapted from Blijlevens & Sonis (2007).

The purpose of this article is to review the published literature on the efficacy of palifermin in the HSCT setting, to present arguments and theories proposed by researchers of postapproval studies examining the efficacy of palifermin, and to make evidence-based recommendations for the use of palifermin in the HSCT setting.

## Literature Review

A literature search was conducted to identify articles that specifically addressed the efficacy of palifermin in the HSCT setting. Articles were obtained from PubMed and Ovid Medline databases using the following search terms: "palifermin", "keratinocyte growth factor (KGF)", "oral mucositis", and "hematopoietic stem cell transplant." The literature search resulted in three randomized controlled trials and six retrospective studies examining the efficacy of palifermin within the HSCT setting. Articles addressing the efficacy of palifermin outside the HSCT setting were excluded.

The literature contains limited published research findings regarding the use of palifermin to prevent and decrease the incidence and severity of oral mucositis in the HSCT setting. The multicenter, placebo-controlled, double-blind, randomized phase III trial conducted by Spielberger et al. (2004) was a landmark study that strongly supported palifermin efficacy in patients undergoing autologous stem cell transplant with a total-body irradiation (TBI)-based conditioning regimen. Results of this study led the US Food and Drug Administration (FDA) to approve this drug in the hematopoietic stem cell transplant setting in 2004.

The Spielberger et al. (2004) study enrolled 212 patients with hematologic malignancies who were undergoing autologous stem cell transplant after a conditioning regimen of fractionated TBI (total dose 12 Gy), high-dose etoposide, and cyclophosphamide. Patients were randomly assigned to receive palifermin (n = 106) 60 µg/kg/day or placebo (n = 106) intravenously for 3 consecutive days before the initiation of TBI and for 3 consecutive days following infusion of autologous stem cell transplant.

**Figure 2:**
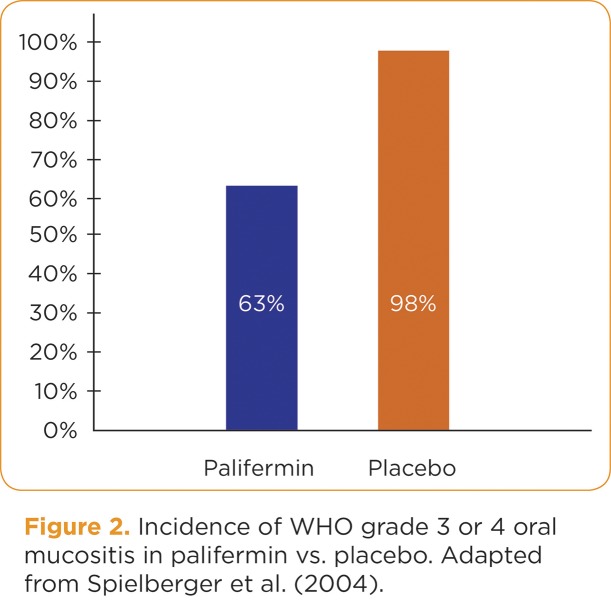
Figure 2. Incidence of WHO grade 3 or 4 oral mucositis in palifermin vs. placebo. Adapted from Spielberger et al. (2004).

**Figure 3:**
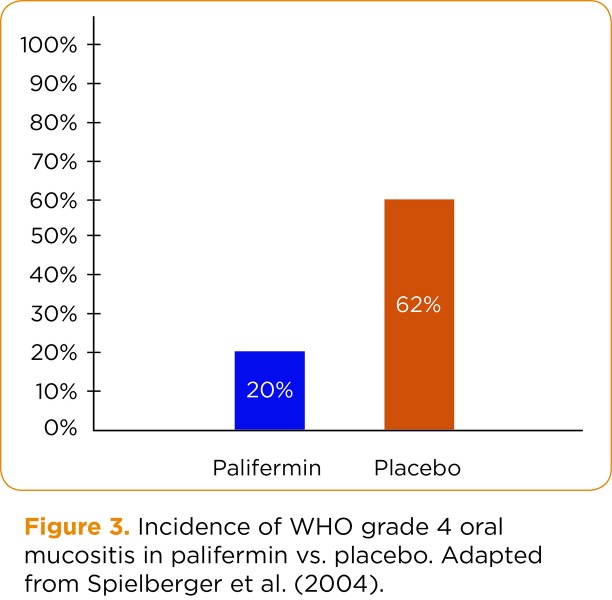
Figure 3. Incidence of WHO grade 4 oral mucositis in palifermin vs. placebo. Adapted from Spielberger et al. (2004).

The incidence of World Health Organization (WHO) grade 3 or 4 OM (see Table 1) was significantly reduced in the palifermin group compared with placebo (63% vs. 98%; * p* < .001; Figure 2). Among patients with this degree of OM, the median duration of OM was only 6 days in the palifermin group compared with 9 days for placebo * p* < .001). In addition, compared with placebo, palifermin was associated with significant reduction in the incidence of WHO grade 4 OM (20% vs. 62%; * p* < .001); see Figure 3. The incidence of use of TPN was also reduced for patients in the palifermin group (31% vs. 55%; * p* < .001). Compared with placebo, palifermin reduced patient-reported mouth and throat soreness by 38% (* p* < .001); palifermin also reduced limitations in functional activities such as drinking, eating, swallowing, talking, and sleeping (Stiff et al., 2006).

**Table 1:**
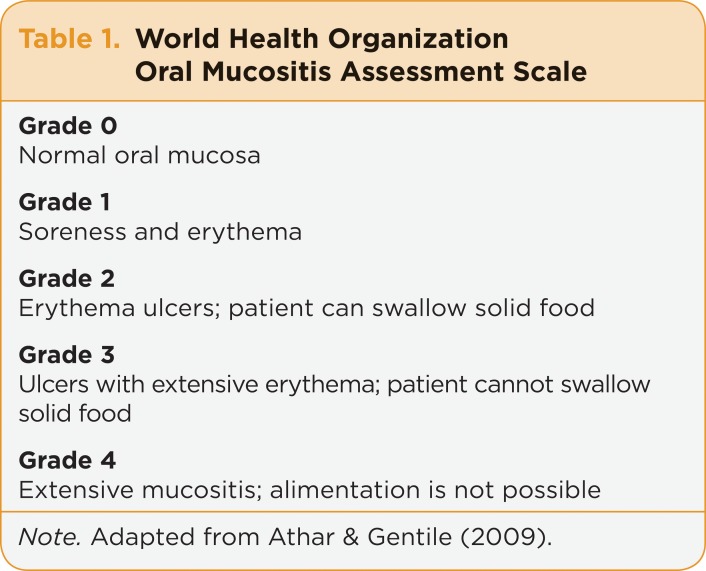
Table 1. World Health Organization Oral Mucositis Assessment Scale

A number of postapproval studies (mostly retrospective) were conducted to further evaluate palifermin efficacy in the HSCT setting using various conditioning regimens with or without TBI (see Table 2 on page 93). A retrospective study by Horsley, Bauer, Mazkowiack, Gardner, & Bashford (2007) included a series of 59 patients undergoing HSCT after receiving non–TBI-based myeloablative conditioning regimens. This study compared a palifermin group (n = 32) with a matched historical control (n = 27) who received standard treatments for management of OM. Palifermin was given 60 µg/kg/day for 3 doses prior to myelotoxic therapy and 3 doses after stem cell transplant, as recommended for postmarketing use. This study demonstrated significant reduction in severe OM (WHO grade 3 or 4) in the palifermin group (13% vs. 48%; * p* = .003). In addition, the palifermin group experienced significant reduction in swallowing problems (* p* = .044) and the number of nutrition-impact symptoms (4.9 vs. 6.0; * p* = .003). These findings are consistent with those of Stiff et al. (2006), who also reported significant improvements in swallowing, eating, and drinking in patients receiving palifermin compared with a control group.

**Table 2:**
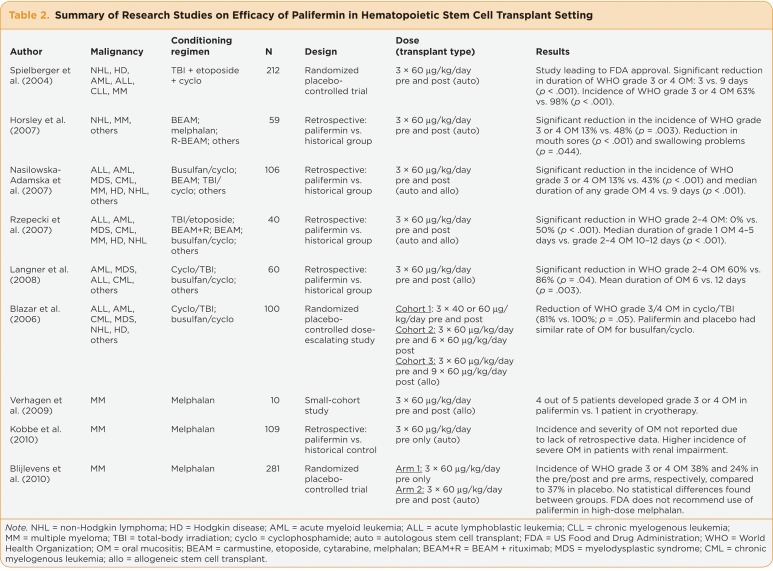
Table 2. Summary of Research Studies on Efficacy of Palifermin in Hematopoietic Stem Cell Transplant Setting

In 2007, another retrospective trial evaluated the efficacy of palifermin in 53 patients undergoing either autologous (54.8%) or allogeneic transplant (45.2%) with or without TBI-based regimens. The incidence, severity, and duration of OM were compared with a historical matched group of patients (n = 53). There was a significant reduction in WHO grade 3 or 4 OM (13% vs. 43%; * p* < .001) and shorter mean duration of grades 1–4 OM (4 vs. 9 days; * p* < .001) in the palifermin group. The incidence of the use of oral, transdermal, and parenteral opioid analgesics (24% vs. 64%; * p* < .001) and TPN (11% vs. 45%; * p* < .001) was also reduced in the palifermin group (Nasilowska-Adamska et al., 2007).

Rzepecki et al. (2007) reported data on patients receiving autologous (n = 11, 55%) or allogeneic (n = 9, 45%) stem cell transplant (from an HLA-identical sibling) with or without TBI-based conditioning regimens for various hematologic cancers. The palifermin group (n = 20) was retrospectively compared with a matched historical control (n = 20). Treatment with palifermin was associated with significant reduction in WHO grade 2–4 OM (0% vs. 50%; * p* < .001). In the palifermin group, 30% of patients developed grade 1 OM with a median duration of 4 to 5 days vs. 10 to 12 days of grade 2–4 OM in the control group (* p* < .001). Moreover, none of the patients in the palifermin group received opioid analgesics or TPN. In the control group 55% of patients required TPN support.

Langner and colleagues (2008) conducted a multicenter trial in which they enrolled 30 patients undergoing matched related donor (MRD) or matched unrelated donor (MUD) allogeneic stem cell transplant for leukemia. They were treated with palifermin and retrospectively compared to a matched control group (n = 30). Two groups were well matched and balanced, with the exception of TBI-based regimens, which was higher in the control group (77% vs. 100%). The incidence of WHO grades 2–4 OM was significantly reduced in the palifermin group (60% vs. 86%; * p* = .04). The mean duration of OM was 6 vs. 12 days (* p* = .003) in favor of palifermin-treated patients. Furthermore, the mean duration of TPN support was also significantly reduced from 26 days in the control group to 16 days in the palifermin group (* p* = .002).

Blazar et al. (2006) reported findings of a randomized, double-blind, placebo-controlled dose-escalating study that compared a palifermin group (n = 69) with matched placebo controls (n = 31) undergoing allogeneic stem cell transplant. Patients were conditioned with cyclophosphamide and fractionated TBI (CY/TBI) or busulfan and cyclophosphamide (Bu/Cy) along with methotrexate and a calcineurin inhibitor (cyclosporine A or tacrolimus) for graft-vs.-host disease (GVHD) prophylaxis. Patients were divided into 3 cohorts and received 6 to 12 doses of palifermin or placebo. The primary objective of this study was to determine the safety and tolerability of palifermin. Secondary objectives were to determine overall survival, the incidence and severity of acute GVHD, and time to marrow engraftment, as well as the incidence, severity, and duration of OM.

Palifermin was associated with a reduced incidence of WHO grade 3 or 4 OM (81% vs. 100%; * p* = .05) and mean severity (2.4 vs. 3.1; * p* <. 05) for patients conditioned with the Cy/TBI regimen. However, in patients conditioned with the less mucotoxic Bu/Cy regimen, the palifermin and placebo groups had similar rates of WHO grade 3 or 4 OM (44% vs. 50%) and mean severity (2 vs. 2.5). Researchers of this study concluded that overall, palifermin was safe in the allogeneic stem cell transplant setting and showed beneficial effects of palifermin with a TBI-based conditioning regimen. No significant differences were found between the palifermin and placebo groups in terms of time to engraftment, incidence of acute GVHD, or survival.

## Efficacy of Palifermin With High-Dose Melphalan as a Conditioning Regimen

**Figure 4:**
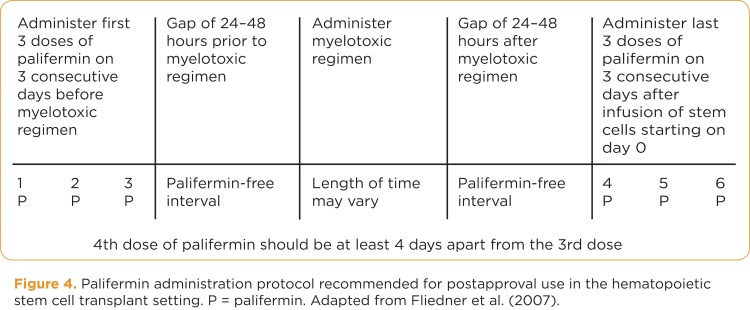
Figure 4. Palifermin administration protocol recommended for postapproval use in the hematopoietic stem cell translant setting. P = palifermin. Adapted from Fliendner et al. (2007).

High-dose melphalan is the most common conditioning regimen used for treatment of multiple myeloma (MM) followed by autologous HSCT (Vogl et al., 2010). Based on the safety and efficacy results of the pivotal study by Spielberger et al. (2004), a strict administration schedule for palifermin is recommended for postapproval use (see Figure 4). However, the dosing intervals and frequency of palifermin recommended for postapproval use have been debated as inappropriate for less mucotoxic and single-agent regimens such as high-dose melphalan (Blijlevens et al., 2010; Verhagen, Wondergem, & Visser, 2009). Research studies implementing the recommended palifermin dosing schedule for postapproval use in the setting of high-dose melphalan have resulted in adverse events and a lack of clinical efficacy.

Review of the literature on the safety and efficacy of palifermin in the high-dose melphalan setting showed one small-cohort study, one retrospective study, and one randomized controlled trial with inconsistent findings. In the small-cohort study, 10 patients were treated for MM with high-dose melphalan (total of 200 mg/m^2^) given over 2 consecutive days followed by autologous stem cell transplant. Five patients received cryotherapy (chewing on ice), and five patients were scheduled to receive palifermin based on the dosing intervals recommended for postapproval use.

Four of the five patients in the palifermin group suffered from WHO grade 3/4 OM compared with only one patient in the non-palifermin group. In the palifermin group, greater use of opioids and less oral intake was also reported. Moreover, palifermin had to be stopped prematurely after three doses in three patients because of skin toxicities and facial swelling; severe swelling of the oral mucosa and neck region occurred after the fourth dose in another patient, indicating possible palifermin toxicity or overdose (Verhagen, Wondergem, & Visser, 2009).

**Figure 5:**
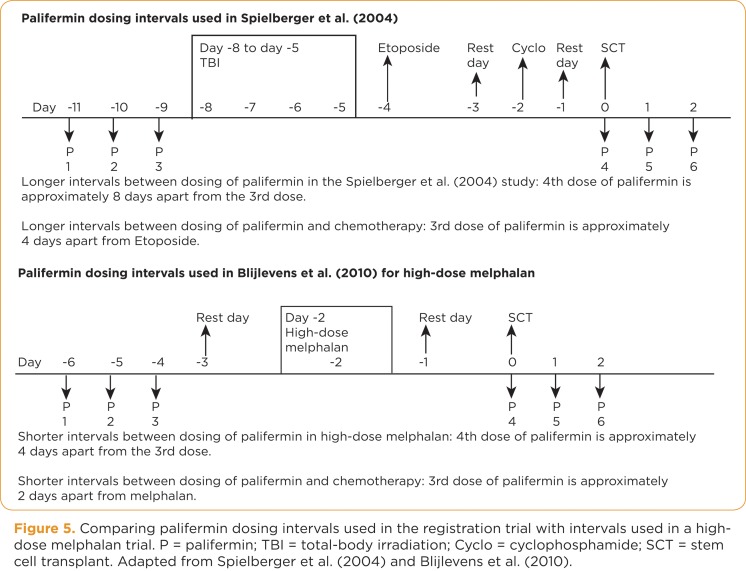
Figure 5. Comparing palifermin dosing intervals used in the registration trial with intervals used in a high-dose melphalan trial. * p* = palifermin; TBI = total-body irradiation; Cyclo = cyclophosphamide; SCT = stem cell transplant. Adapted from Spielberger et al. (2004) and Blijlevens et al. (2010).

Authors of this small study argued that compared with palifermin dosing intervals used in the registration study by Spielberger et al. (2004), the dosing interval is shorter in the high-dose melphalan conditioning regimen. They highlighted that shorter intervals between palifermin dosing most likely created a cumulative effect that led to overdose and necessitated the discontinuation of palifermin. In addition, it is also important to note that the dosing interval between palifermin and chemotherapy is reduced in the high-dose melphalan regimen (see Figure 5). Shorter intervals between palifermin dosing and chemotherapy have been speculated to increase the severity of OM.

In one of the preapproval, placebo-controlled, randomized clinical trials, increased severity and duration of OM were observed when palifermin was administered within 24 hours of chemotherapy. It was theorized that the administration of palifermin and chemotherapy within close intervals generated a counteracting effect. In the time period immediately after chemotherapy, epithelial cells stimulated by palifermin are highly sensitive to the damaging effects of chemotherapy, which contributes to increased severity of OM (US FDA, 2011). Verhagen et al. (2009) further argued that the palifermin dosing in the Spielberger et al. (2004) study was designed for a highly mucotoxic chemoradiotherapy regimen. In less mucotoxic and single conditioning-based agents such as high-dose melphalan, widening the dosing intervals or reducing the frequency of palifermin might shed light on the potential benefits of palifermin.

The theory of reducing the dosing frequency of palifermin based on the mucotoxic potential of the conditioning regimen was examined by Kobbe et al. (2010) in a retrospective study. This study enrolled 67 patients with MM who received 3 consecutive doses of palifermin (60 µg/kg/day) approximately 2 days prior to melphalan infusion (200 or 140 mg/m^2^ for creatinine clearance < 50 mL/min). No additional palifermin was given after the infusion of stem cells. One of the aims of this study was to determine whether three doses of palifermin could reduce the toxicities associated with oral mucositis such as length of hospital stay, infections requiring antibiotics, and the use of narcotic analgesics and TPN.

Results of this study showed that the palifermin group spent significantly fewer days in the hospital compared with the historical group (* p* < .05) and also received fewer days of parenteral opioid analgesia (* p* < .05). No differences were found in the number of days of IV antibiotic use. Results on days of TPN use were confounded, as patients were started on TPN for reasons other than OM, such as nausea and vomiting, which was reported as being the reason for a significant number of patients. In addition, the incidence and severity of OM were not compared between the palifermin and the historical group due to a lack of retrospective data on these parameters.

A subanalysis of the palifermin group, including patients with normal renal function and patients with creatinine clearance < 50 mL/min, revealed a higher incidence of severe OM in patients with impaired renal function (16% vs. 64%; * p* < .002). The reason for this difference between the palifermin groups is not clear. In contrast to the study by Verhagen et al. (2009), three doses of palifermin approximately 2 days prior to high-dose melphalan were well tolerated by most patients, and no significant toxicities were reported in this study.

In a postapproval, randomized, placebo-controlled, double-blind study, the theory of modifying palifermin dosing frequency was further evaluated in patients with MM receiving high-dose melphalan (200 mg/m^2^) followed by autologous stem cell transplant (Blijlevens et al., 2010). A total of 281 patients were randomized to 3 arms: the pre/post arm (n = 115) received 3 doses of palifermin before and 3 doses after melphalan infusion; the pre arm (n = 109) received only 3 doses of palifermin before melphalan, and the placebo arm (n = 57). The incidence of WHO grade 3 or 4 OM was 38% and 24% in the pre/post and pre arms, respectively, compared to 37% in the placebo arm. There were no significant differences noted between either of the palifermin arms and the placebo arm. The pre arm showed better results numerically and also experienced a more favorable safety profile as compared to the pre/post arm.

Researchers concluded that differences in the mucotoxicity profile of melphalan and shorter intervals between palifermin pre and post dosing compared to the registration trial might explain the lack of significant differences between groups in this study (Blijlevens et al., 2010).

Based on the results of this randomized controlled trial, the FDA issued a new drug label in November 2011 recommending against the use of palifermin in the setting of high-dose melphalan as a conditioning regimen due to the lack of clinical efficacy (US FDA, 2011). Nonetheless, the favorable toxicity profile seen with a total of three doses of palifermin observed by Kobbe et al. (2010) and Blijlevens et al. (2010) adds strength to the theory that frequency of palifermin dosing should be modified based on the mucotoxicity of the conditioning regimen to prevent overdose and adverse effects of palifermin.

## Efficacy of Palifermin When Administered Concomitantly With Methotrexate

According to the FDA, palifermin should not be administered within 24 hours before, during the infusion, or within 24 hours after administration of myelotoxic chemotherapy, as it may lead to an increased severity and duration of oral mucositis. Hence, the use of methotrexate (given on days 1, 3, 6, and 11) as GVHD prophylaxis in the allogeneic stem cell transplant setting concomitantly with palifermin (given on days 0, 1, and 2) has been theorized to increase the severity of OM or offset the beneficial effects of palifermin (Blazar et al., 2006; McDonnell & Lenz, 2007; van der Velden, Herbers, & Blijlevens, 2009).

However, in two retrospective studies and one randomized controlled trial, the palifermin group experienced a reduced incidence and duration of OM despite concomitant administration of methotrexate (Blazar et al., 2006; Langner et al., 2008; Nasilowska-Adamska et al., 2007). In the randomized placebo-controlled, dose-escalating study by Blazar et al. (2006), investigators observed no interaction between palifermin and methotrexate in the severity of OM or tolerance of all four doses of methotrexate.

## Economic Impact of Palifermin

Elting and colleagues (2007) retrospectively analyzed the economic impact of palifermin on outcomes such as incidence of febrile neutropenia, infection, and use of TPN. This study compared the estimated total hospital costs incurred by patients who received palifermin with those incurred by patients who received placebo in the registration study by Spielberger et al. (2004). The average sale price of palifermin is reported to be approximately $8,250 per patient (six doses). Investigators hypothesized that reduction in costly interventions associated with severe OM by palifermin might offset the acquisition cost of the drug or lead to cost savings. Results of this study indicated a nonsignificant mean savings of $3,595 per patient in the palifermin group after accounting for the additional price of the drug. Nonsignificant mean savings indicated that the use of palifermin in this patient population was cost-neutral. Results demonstrated that the acquisition cost of palifermin was offset by a reduction in the frequency of adverse outcomes, particularly from significant lower utilization of TPN. From both the clinical and economic perspectives, investigators concluded that use of palifermin is justified in patients with hematologic malignancies receiving high-dose TBI-based conditioning regimens.

Although the use of palifermin offers a favorable economic profile for high-dose TBI-based regimens, investigators cautioned that the cost profile may not be as favorable in the less mucotoxic or non–TBI-based regimens where the potential for severe OM is much lower. Elting et al. (2007) emphasized that the economic profile of palifermin is affected by many factors, including transplant type, mucotoxicity of treatment regimen, and patient population. Therefore, the economic impact of palifermin in these settings requires further investigation.

## Side Effects

The most common adverse reactions attributed to palifermin are skin and oral toxicities. In the pivotal study by Spielberger et al. (2004), skin rash, itching, paresthesia mostly localized to the oral region, thickening of the tongue, and taste alterations were reported more frequently in the palifermin group. All of these events were mild to moderate in severity, transient, and did not result in discontinuation of palifermin. In addition, a transient asymptomatic increase in serum amylase and lipase levels was observed more frequently in the palifermin group than with placebo, which returned to near baseline values by the day of transplantation.

## Discussion

The efficacy of palifermin in high-dose TBI-based conditioning regimens is well established and consistently supported by Spielberger et al. (2004) phase II and III randomized controlled trials. However, extrapolation of palifermin dosage and dosing frequency used in the registration study for non-TBI or less mucotoxic conditioning agents has been debated. The debate is that non–TBI-based regimens are far less toxic than high-dose TBI-based regimens. Therefore, palifermin dosing used in the registration study is not appropriate for non–TBI-based conditioning regimens.

Researchers of postapproval studies have theorized that for less mucotoxic or non–TBI-based regimens, reducing the dosage or frequency of palifermin might reveal a more justified risk-benefit ratio (Blijlevens et al., 2010; Kobbe et al., 2010; Verhagen, Wondergem, & Visser, 2009). For instance, in a randomized controlled trial using high-dose melphalan as a conditioning regimen, the group that received a total of three doses of palifermin demonstrated a better side-effect profile and also better results numerically than did the group that received six doses of palifermin or placebo (Blijlevens et al., 2010). This result adds strength to the theory of modifying the frequency of palifermin administration based on the mucotoxicity potential of the conditioning regimen to prevent adverse effects and generate the greatest possible clinical benefits.

In addition, the postapproval protocol for palifermin dosing intervals has also been questioned. The approved protocol for palifermin dosing is not completely parallel to that used in the registration study. The intervals between palifermin dosing and also between palifermin and chemotherapy were longer in the registration study than what is currently recommended for postapproval use. Researchers of postapproval studies have theorized that shorter intervals between palifermin dosing may produce a cumulative effect that could lead to overdose and adverse events. In addition, shorter dosing intervals between palifermin and chemotherapy may cause newly developed oral mucosal cells stimulated by palifermin to be more sensitive to chemotherapy-induced damage, resulting in severe OM or offsetting the beneficial effects of palifermin (Blijlevens et al., 2010; Vadhan Raj et al., 2010; Verhagen, Wondergem, & Visser, 2009).

These theories merit attention, as it has been reported that the biological and therapeutic effects of palifermin persist after the drug level has dissipated. Moreover, it is not completely clear how long the biological effects can last. Clarification of this issue will help to optimize the dosing schedule of palifermin to achieve clinical benefits and prevent adverse events (Health Canada, 2007). Future randomized trials focusing on widening the dosing intervals of palifermin would be helpful to determine if this could have any impact on the incidence and severity of OM for less mucotoxic or non–TBI-based regimens.

Modifying the dosage and dosing frequency of palifermin for less mucotoxic agents has been investigated in the non–stem cell transplant setting, demonstrating beneficial effects. In a small-cohort study, 10 patients treated with high-dose methotrexate for acute lymphoblastic leukemia and lymphoma developed severe OM in the first cycle. All 10 patients received palifermin, with dosage ranging from 30 µg to 60 µg/kg/day (total of 6 doses) in the subsequent cycle. Only 2 patients suffered from severe OM compared to all 10 in the first cycle (Schmidt et al., 2008).

In a randomized, placebo-controlled trial, a single dose of palifermin (180 µg/kg) was given 3 days prior to a doxorubicin-based regimen in patients with sarcoma. This study showed significant reduction in severe OM in the palifermin group. Authors of this study highlighted that a single dose of palifermin given 3 days before the chemotherapy eliminates the risk of administration of chemotherapy and palifermin within close intervals and also offers convenience for patients (Vadhan Raj et al., 2010). Results of these two studies offer encouragement and opportunity to investigate reduced dosage and frequency of palifermin in the HSCT for less mucotoxic agents. Reducing the dosage or frequency also reduces the cost of drug acquisition, rendering use of palifermin economically feasible.

The incidence and severity of OM vary significantly across different conditioning regimens. The incidence of WHO grade 3 or 4 OM is approximately 20% for the BEAM regimen, 50% for the high-dose melphalan regimen, and close to 95% in high-dose TBI-based regimens (European Medicine Agency, 2005). Therefore, cost-effectiveness and clinical benefits of palifermin among various conditioning regimens have also been debated (Elting et al., 2007). Approximately 20% of patients undergoing the BEAM regimen suffer from severe OM, while the other 80% may develop mild OM or none at all. In this case, use of palifermin is unjustified from both an economic and a clinical standpoint.

## Future Research

To clinically and economically justify the use of palifermin in the HSCT setting, the FDA issued a new drug label in November 2011 restricting the use of palifermin for conditioning regimens with an anticipated incidence of OM of ≥ grade 3 OM by WHO classification in the majority of patients (US FDA, 2011). Future research should be aimed at identifying individuals who are at risk for developing severe OM to justify the use of palifermin for less mucotoxic regimens. In one of the prospective studies, being female, having an extensive history of chemotherapy treatments, and having a prior incidence of OM were reported as potential risk factors for the development of OM after the BEAM conditioning regimen (Strobel, Bauchmuller, Ihorst, & Engelhardt, 2006). Continued research such as this could guide clinicians in identifying vulnerable patients who could potentially benefit from palifermin treatment in advance and allow for more favorable clinical and economic outcomes (Weigelt, Haas, & Kobbe, 2011).

Randomized controlled trials examining the efficacy of palifermin in the HSCT setting are scarce. Although most postapproval retrospective studies in the HSCT setting have demonstrated significant reduction in severe OM in the palifermin group, it is important to note the limitations of these studies. First, sample sizes in these studies were small and not randomly selected. Second, data on the incidence and severity of OM were collected retrospectively via medical charts, which can introduce bias. Finally, patients receiving various conditioning regimens were included and analyzed together. This design method fails to demonstrate the efficacy of palifermin for an individual conditioning regimen. Since the incidence of severe OM varies significantly between different conditioning regimens, it is important that research studies testing the efficacy of palifermin are randomized and designed to include one conditioning regimen between groups. This would permit clear analysis of clinical efficacy and determine economic feasibility of palifermin for the regimen used in the study.

Due to a lack of clinical efficacy observed in a randomized controlled trial, the FDA does not recommend the use of palifermin in the setting of high-dose melphalan. Use of methotrexate as GVHD prophylaxis in an allogeneic setting concomitantly with palifermin is theorized to increase the severity of OM. However, in two retrospective studies and one randomized controlled trial, reduced incidence and severity of OM were observed despite concurrent use of palifermin and methotrexate. Based on the results of these studies, administration of palifermin concomitantly with methotrexate appears safe.

## Conclusions

The efficacy of palifermin for high-dose TBI-based conditioning regimens is clear and strongly supported by randomized controlled trials. Use of palifermin within these contexts offers both clinical benefits and a favorable economic profile. However, many questions remain regarding the efficacy of palifermin in non-TBI or less mucotoxic regimens. Future research on risk prediction to identify vulnerable individuals at risk for developing severe OM is warranted. Use of palifermin for high-risk individuals may offer favorable clinical and economic outcomes. Another area of research is to reduce palifermin dosage or frequency, and to modify palifermin dosing intervals in a randomized study to determine the impact of palifermin on the incidence and severity of OM for non-TBI or less mucotoxic conditioning regimens.
